# Probabilistic Method to Fuse Artificial Intelligence-Generated Underground Utility Mapping

**DOI:** 10.3390/s24113559

**Published:** 2024-05-31

**Authors:** Kunle Sunday Oguntoye, Simon Laflamme, Roy Sturgill, Daniel A. Salazar Martinez, David J. Eisenmann, Anne Kimber

**Affiliations:** 1 Department of Civil, Construction and Environmental Engineering, Iowa State University, Ames, IA 50011, USA; laflamme@iastate.edu (S.L.);; 2 Department of Electrical and Computer Engineering, Iowa State University, Ames, IA 50011, USA; 3 Department of Materials Science and Engineering, Iowa State University, Ames, IA 50011, USA

**Keywords:** utility mapping, artificial intelligence, subsurface utility engineering, data interpretation, knowledge fusion

## Abstract

Utility as-built plans, which typically provide information about underground utilities’ position and spatial locations, are known to comprise inaccuracies. Over the years, the reliance on utility investigations using an array of sensing equipment has increased in an attempt to resolve utility as-built inaccuracies and mitigate the high rate of accidental underground utility strikes during excavation activities. Adapting data fusion into utility engineering and investigation practices has been shown to be effective in generating information with improved accuracy. However, the complexities in data interpretation and associated prohibitive costs, especially for large-scale projects, are limiting factors. This paper addresses the problem of data interpretation, costs, and large-scale utility mapping with a novel framework that generates probabilistic inferences by fusing data from an automatically generated initial map with as-built data. The probabilistic inferences expose regions of high uncertainty, highlighting them as prime targets for further investigations. The proposed model is a collection of three main processes. First, the automatic initial map creation is a novel contribution supporting rapid utility mapping by subjecting identified utility appurtenances to utility inference rules. The second and third processes encompass the fusion of the created initial utility map with available knowledge from utility as-builts or historical satellite imagery data and then evaluating the uncertainties using confidence value estimators. The proposed framework transcends the point estimation of buried utility locations in previous works by producing a final probabilistic utility map, revealing a confidence level attributed to each segment linking aboveground features. In this approach, the utility infrastructure is rapidly mapped at a low cost, limiting the extent of more detailed utility investigations to low-confidence regions. In resisting obsolescence, another unique advantage of this framework is the dynamic nature of the mapping to automatically update information upon the arrival of new knowledge. This ultimately minimizes the problem of utility as-built accuracies dwindling over time.

## 1. Introduction

Accidental strikes of underground utilities during construction and other digging activities are common and can lead to serious injuries and substantial economic losses [1,2]. The accurate identification and location of buried utilities is a critical step in ensuring worker safety and utility integrity [3]. Underground utility investigations are typically initiated using as-built or as-planned records and then utility locating services, such as those from Subsurface Utility Engineering (SUE) providers [4]. As-built or as-planned records are not always readily available and may be inaccurate [5]. The popular One-Call system acts as a clearinghouse to notify utility companies of imminent construction activity and puts them on notice to indicate where their facilities are located with ground paint and flagging. Utility companies often use a third-party service for this activity and the resulting markings are known to be inaccurate. The One-Call system is useful in promoting utility damage prevention awareness, yet may also yield incomplete utility information as it commonly does not include service lines or out-of-service utilities [6].

Recent advances in computer vision and artificial intelligence (AI) present an alternative solution that automatically generates utility maps. The approach makes inferences about the location of underground utilities from observations of aboveground appurtenances, such as electrical meters and manholes. The method estimates the existence, location, and path of possible utilities associated with the detected appurtenances. The collection of inferences becomes an AI-generated utility map. AI-generated utility mapping is an existing technique that has been used to create maps for fluid-carrying utilities through the detection and connection of manholes and similarly infer utility alignments from observations of electric transformers and poles. The underground utility locations are inferred by predicting patterns based on established fuzzy logic rules [7,8,9]. Compared to other methods, this automated methodology of utility mapping using advanced data fusion algorithms is non-intrusive, and, particularly compared to ground penetrating radar (GPR) [10] methods, faster and less expensive. The method can also rely on existing datasets, for example using data from Google Maps, or leveraging advances in autonomous aerial and ground vehicles for data collection [11]. However, AI-generated utility maps using computer vision alone may not be sufficient to determine the exact location of underground utilities and must be paired with a knowledge database or other investigation methods to increase the level of location confidence [12].

An important aspect of such a knowledge database is the availability of information such as as-built or as-planned maps. When available, these maps can be used to build confidence or refine the location of utilities within the AI-generated map. However, the fusion of these data with the AI-based generated maps can present challenges in cases where the mapping finds conflicting utility paths [13]. There is a significant likelihood for these challenges to occur because as-built/as-planned maps are often inaccurate and would therefore not exhibit the same spatial information as that from an AI-generated map. This work shows that the fusion of both maps can yield probabilistic mapping that conveys confidence bands for the most likely locations of utilities within a specific area, with the width of these bands being a function of the similarities and disparities between available data.

There have been previous efforts to fuse knowledge with surveyed data. For instance, Zhou et al. [14] developed a probabilistic mapping model to maximize the likelihood of fitting a utility pipeline to existing knowledge by combining data from utility records, locations of manhole covers, and GPR investigation. Lanka et al. [8] used fuzzy logic approximate reasoning to identify utility location confidence through the combination of manhole locations, subject matter expertise, and GPR investigation. Chen and Cohn [15] integrated information from similar sources [8] using Bayesian data fusion techniques to estimate the probability that any given point of interest contains a buried utility. Li et al. [16] combined a global positioning system-aided GPR survey with preexisting knowledge of utility depth from excavation efforts to understand the limitations of GPR equipment through a comparison of sensor outputs with ground truth data, thus enabling the creation of an uncertainty-aware 3D model. Other works [5,17,18] fused utility data sources using similar probabilistic approaches similar to the aforementioned studies [8,14,15].

A key limitation of the majority of the previous works cited is the reliance on labor-intensive field data collection, such as the collection of GPR data and surveying appurtenances, which can be significantly costly to achieve [19]. In this paper, we propose fusing information generated through the use of AI and available data such as as-built or as-planned records. This method can be viewed as an initial stage of a broader utility investigation. The data provided in fusing such knowledge lead to a refined and confidence-infused map from which one can minimize the more expensive stages of a utility investigation. The approach consists of sequentially (1) overlaying available data; (2) identifying similarities and conflicts; (3) pruning information; (4) producing a probabilistic utility map; and (5) recommending actions to reduce uncertainties and refine the utility map. The methodology is demonstrated using field data from a site in Ames, IA, for which an as-built utility record was available.

The rest of the paper proceeds with a discussion of the demonstration site and the creation of the AI-generated utility map. Next, the data fusion methodology is presented, and a demonstration is provided of how the AI-generated map can be fused with the available utility records. Finally, conclusions are drawn and include a discussion on potential gaps that could be addressed in future research efforts.

## 2. AI-Generated Utility Map

**Figure 1:**
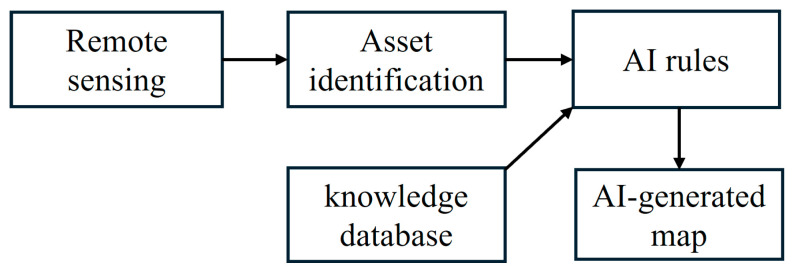
AI-generated map flowchart.

The AI-generated utility map is created using the algorithm diagramed in Figure 1. Measurements collected from remote sensing, typically consisting of high-resolution pictures or satellite imagery, are used to identify aboveground assets. Such identification can be automated using computer vision algorithms. Aboveground assets are associated with utility classes and linked using AI rules to generate a map. The AI rules can be inherited from a knowledge database, as described in [12], for example using information entered by expert agents, sparsed from utility codes, inferred from topologies, etc. 

### 2.1. Selected Site and Available As-Built Map

**Figure 2:**
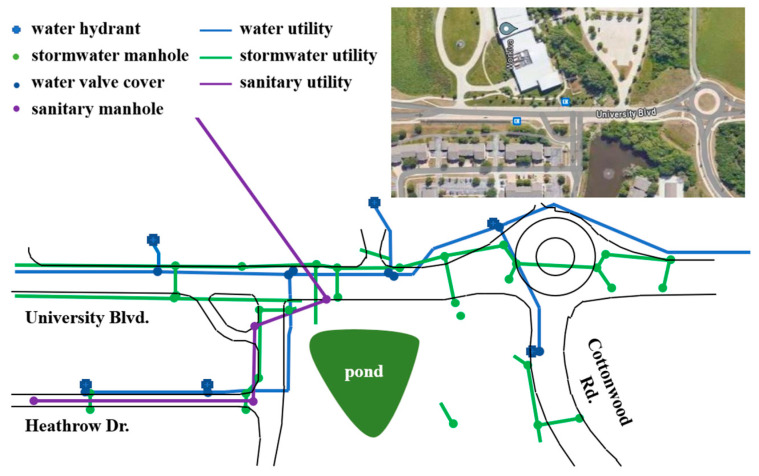
Existing as-built map and aerial image of the area of interest (inset).

The utility as-built map of the demonstration site shown in Figure 2, is located in Ames, Iowa, within Iowa State University’s Research Park. The site was selected due to its low traffic volume and the availability of a utility as-built map. From discussions with Iowa State University’s Facilities Planning and Management personnel, the as-built map contains the most up-to-date information available but may include inaccuracies. The as-built map contains information on potable water, storm sewer and drainage, and sanitary sewer utilities; electricity, gas, and telecommunication utilities are not included for security purposes. The given level of uncertainty in the as-built map combined with the known lack of information provides an excellent opportunity to evaluate the proposed utility map fusion methodology.

### 2.2. AI-Generated Utility Map

To produce the initial AI-generated utility map for the selected site, a comprehensive site survey was completed to identify aboveground appurtenances according to their utility type and collect their geographic location and position relative to other infrastructure. Some appurtenances may belong to multiple asset classes and therefore may require additional effort for identification, such as reading the inscriptions on manholes and valve covers. Hence, the site survey was conducted manually, which also allowed the study to focus on the problem of map information fusion. Information collected using remote sensing is to be integrated into future research efforts.

**Figure 3:**
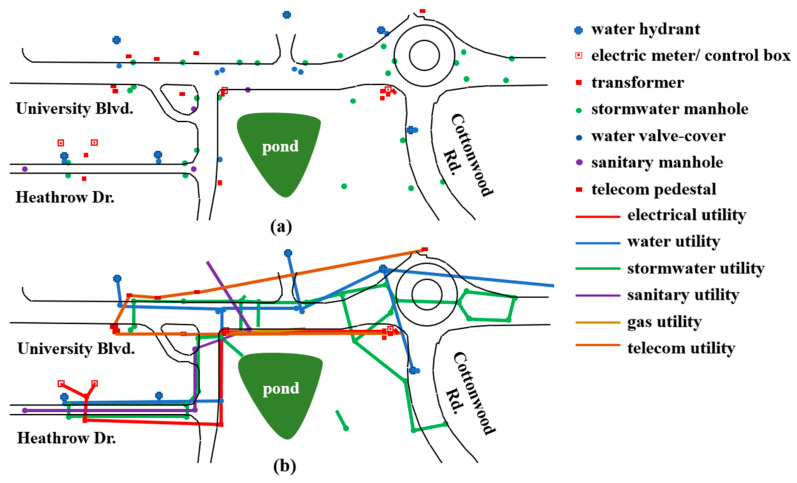
(**a**) Identified aboveground appurtenances and (**b**) AI-generated utility map.

Appurtenances were identified using an approach similar to existing studies [7,8]. Table 1 lists the various appurtenances surveyed within the investigation site, with their location shown in Figure 3a. Next, the connections and orientations of the underground utility assets were inferred using hierarchical deterministic logic rules that directly apply to the identified appurtenances. These rules are organized in groups for each utility class that guide the hierarchical order of inferring the utility asset connections. The rule groups for each utility class include utility proximity, the relationship with nearby roadways, and downstream endpoints in establishing the application order of the rules. The proximity rules establish utility connections between close appurtenances of a similar utility class, the roadway right of way establishes the utility connection orientation, and the downstream endpoints establish the direction of service lines from the main utility. Table 2 lists the hierarchical rules used for the major utility classes and Figure 3b shows the connected appurtenances using these rules.

Evaluating the completeness of these rules is not within the scope of this paper and is left to future work. Instead, they are used to produce an AI-based generated map to demonstrate the proposed data fusion method. In future applications, additional or different rules could be adopted, such as the integration of multi-source knowledge as discussed in [12].

## 3. Utility Location Data Fusion

### 3.1. Process

**Figure 4:**
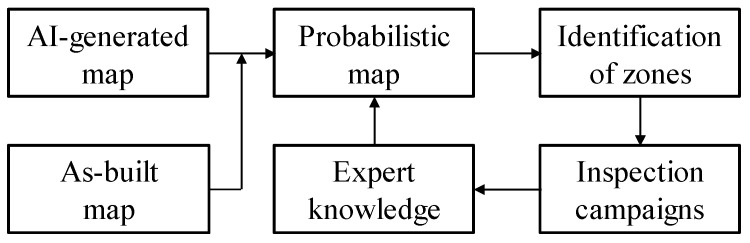
As-built data fusion process.

The utility location data fusion process is illustrated in Figure 4. The AI-generated utility map and the utility as-built (or as-planned) map are overlaid to create a probabilistic map. The probabilistic map shows the location of utilities accompanied by levels of confidence. In common cases, problematic or low-confidence zones that require further refinement are identified. This refinement is conducted through the integration of expert knowledge or utility investigation, such as targeted GPR investigation. The process is iterated until the accuracy of the refined probabilistic map is deemed appropriate for the planned task (e.g., digging).

The fusion of the utility location data (the AI-generated map and the as-built map) occurs using probability distributions. The probability distributions represent the probability of a utility existing in a specific location. A unique distribution is assigned to each data source (e.g., the utility as-built map) being used. For this study, the focus is on two-dimensional mapping and the distributions for each source are assumed to be normal $N(\mu ,\sigma^{2})$ with the orientation of the distribution perpendicular to the utility vector. For the distribution, μ is aligned to the utility location identified in a given data source map, and σ is the standard deviation. Joining the probability distributions of varying data sources for one utility (i.e., the waterline shown in the as-built map joined with the same waterline shown in the AI-generated map) in a two-dimensional overlaid map provides the probabilistic map. The probabilistic map can be refined by pruning areas that are below a desired probability threshold. Again, the scope of this paper is confined to 2D locations of utilities, so the identification of utility depth is a topic for future investigation. The methodology can be extended to include buried depth by augmenting the dimension of the normal distribution and providing three-dimensional probabilistic maps.

In the process of generating the probability distribution for the location of a utility, the standard deviation σ can be adjusted by the user to represent data confidence. A large σ value represents low confidence, and conversely, a small σ value represents a high level of confidence. This value can be estimated using subject matter expertise. For example, the availability of construction photos showing the as-built utility location may result in estimating a low σ, while a utility location based on a dated as-planned map may result in estimating a high σ. These estimates can also be refined based on a review of the data sources, for example, when multiple data sources closely align, a low σ may be estimated.

Next, pairing is necessary for joining the probabilities across data sources. This is where a given utility line section from one source map is assigned a twin from another source map. For this pairing, the orientation of line segments is utilized, along with the utility endpoints, such as manholes and fire hydrants.

**Figure 5:**
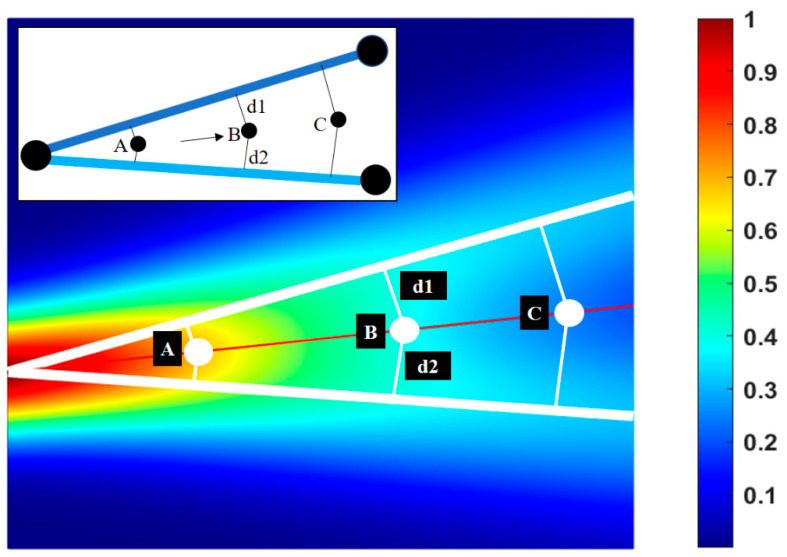
Normalized probability gradient of utility segments of average and equal standard deviation and weight (thick white lines represent the utility lines while the red line represents a mid-line for topological distance calculation).

An example of pairing is shown in Figure 5 (inset), where one line is from the AI-generated map and the other line is from the as-built map, but both lines represent the same utility segment. In this case, pairing is achieved through the identification of a shared endpoint (left-most black circle) and the close proximity of segments moving to the right. Given that the probability distributions run perpendicular to each line, the distance between the segments for a given point is defined as a topological path where $d_{1}+d_{2},$ which runs along the intersection of the distribution lines. Figure 5 shows the joined probability distribution gradient plot varying from 0 to 1. Here, the red color denotes the region of higher confidence, and blue denotes lower confidence.

Another aspect to consider among data sources is prioritization, where one data source could be evaluated as more significant than another. This consideration can be assigned a weighting, where the weights of the data sources used in Figure 5 are assumed to be equal. The weights (w) constitute a second set of user-defined parameters. The summation of weights over the sources for the same utility must sum to 1 in order to create joint probability distribution functions and the probability distribution gradient plot.

### 3.2. Factors Affecting Confidence

The standard deviation σ and source weights *w* inform the degree to which a data source influences the probability gradient for a unique utility segment. These parameters can be refined as more becomes known about the accuracy of a data source through more extensive utility investigations. As shown in Figure 5, the utility segments are evenly weighted with the assumption that the AI-generated map is as accurate as the as-built map. In this case and in most real-world examples, the sources are likely not equivalent in accuracy or prioritization. A user will need to assign initial standard deviations based on the confidence within a given dataset and the prioritization weight based on the confidence across all data sources used.

As initial guidance, the initial standard deviation can be either individually set as a function of the utility’s geometry (e.g., the width of a typical trench) or weights set equally awaiting further knowledge. The weights and standard deviations used within this approach are influenced by many factors including, but not limited to, the following:

Date of a data source: evidence in the literature shows that the accuracy of data sources such as as-built maps decreases as the age of that source increases [21,22,23]. A dated data source may not reflect changes in utilities, terrain, or the surrounding environment that occur over time, making the source obsolete compared to other sources.

Utility rule compliance: data sources may also be constructed based on expectations, such as those forming the basis of the rules followed in constructing the AI-generated utility maps. These rules or expectations are formed from common practices. The AI generation inherently assumes that these utility rules are strictly followed. However, site conditions or other reasons may lead to the rules not being followed, for example, features in close proximity may not always be connected if site conditions prevent this in some way.

Sensor data: different sensors such as GPR and electromagnetic sensors are popularly used to investigate the position of underground utilities. These sensors do have limitations that can constrain their applicability and create inconsistencies in the resulting data. Also, since this type of data are mostly expert-interpreted, human error and bias are possible. An example might include the interference with electromagnetic signals due to overhead electric lines or the presence of nearby metals. Hence, these factors are to be considered in selecting the appropriate weight and standard deviation for the data source.

As-built versus as-planned sources: the nature of the utility records in the knowledge database is crucial in ascertaining the accurate weight. Often, utility construction plans are submitted unaltered as the as-built plans to satisfy permitting requirements. These as-planned utility maps do not record or depict changes in position and orientation that may occur during construction. Inherently, they are less reliable compared to the actual as-built recorded information. Whether there is knowledge available to distinguish the record as an as-built or as-planned record will factor into setting weights and standard deviations for these sources.

Construction variance: most utilities are permitted to be installed within criteria and limits. They may also be confined by policy or procedure to be constructed within such limits. These limits may include certain depths, distances from a roadway, or distances from other utility infrastructure. However, there are records of noncompliance in utility construction, as revealed in the literature reviewed [24]. Thus, there may be uncertainties to consider if there is knowledge of noncompliance associated with a particular utility or data source.

Aboveground asset occlusion: the AI-generated map depends on visual cues such as aboveground assets for its map development. In an event where these assets are obscured by vegetation or man-made features, these accessibility challenges may result in incomplete and inaccurate utility routing.

Ground-truth data: some data sources have a known high level of accuracy and confidence. For instance, the height, depth, and orientation of utility may be concisely derived from open-cut trenches or manhole investigations and would represent the most accurate information in the knowledge database. Therefore, in this case, a low standard deviation would be expected with a weight value approaching 1.0. Human error may present reasoning for less than perfect confidence and accuracy.

There are many other factors that can influence weight and standard deviation assignments. Additionally, discoveries from sensors and other field investigations could result in cases where no other source includes twin-utility segments for this new knowledge. A possible solution to this issue would be a larger standard deviation with no comparison twin. Table 3 lists guidance for users in considering weights and standard deviations based on the knowledge of the utilities.

## 4. Results

The proposed fusion technique was applied to the as-built and AI-generated utility maps for the test site outlined in Section 2. Though this section explores the water utility system from both the as-built and AI-generated maps, the proposed methodology is equally applicable to other utility classes.

**Figure 6:**
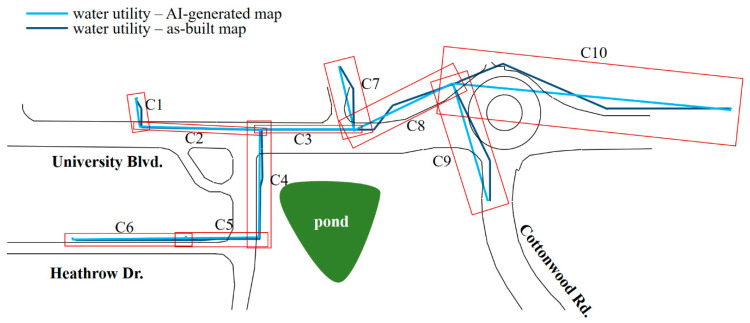
Water utility overlay map subdivided into sections C1–C10.

Figure 6 shows the overlaid map of the water utility system, delineating areas with twin utility segments whose endpoints coincide or are in close proximity (less than 5 m). These bounded areas include segments of strong correlation in the source maps (e.g., C1 to C6) and some with larger disparities between the twin utility segments as observed in C7 to C10.

**Figure 7:**
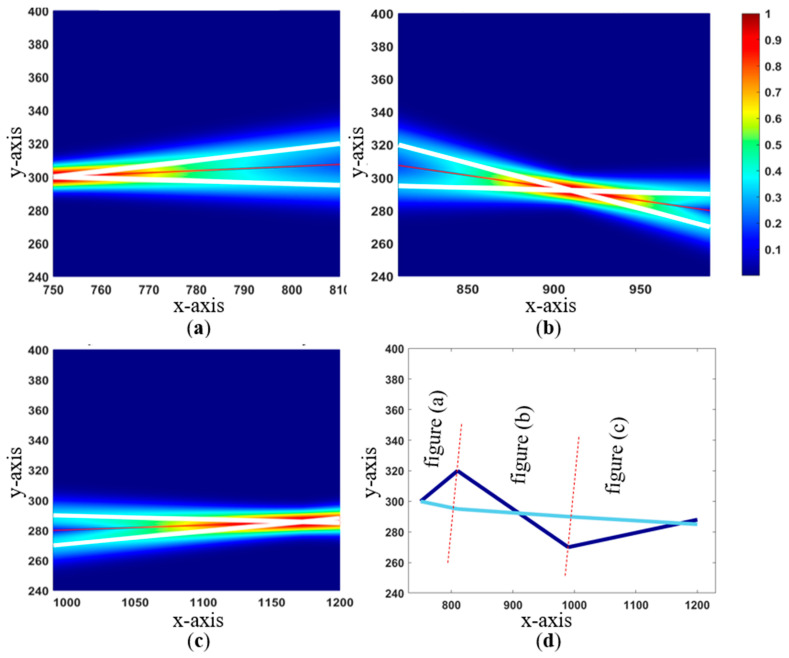
(**a**) C10 subsection (left) probabilistic plot; (**b**) C10 subsection (middle) probabilistic plot; (**c**) C10 subsection (right) probabilistic plot; (**d**) C10 connections with red dashed line indicating subsections.

Area C10 is selected to demonstrate the proposed framework due to the large disparity between the as-built and AI-generated utility maps. In the initial probability plot shown in Figure 7, where the x-axis is set as a function of the first subsection’s AI-generated water utility line, both maps are evenly weighted. However, the as-built map age could not be validated, and the areas have had significant construction activities, including a suspicion that electric and gas utilities might have been added or removed. Therefore, the as-built map should be assigned a lower weight compared to the AI-generated map. The AI-generated map asset identification was manually conducted through an onsite visual inspection with limited physical resources, leading to the possibility of missing some field data. In an initial review, equal weights are used for these two data sources.

**Figure 8:**
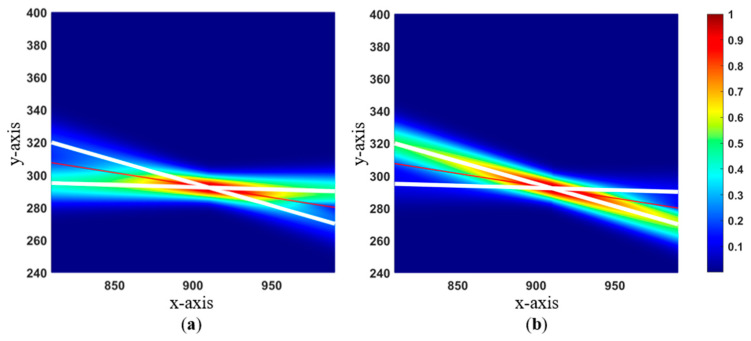
Probabilistic gradient plot upon weight update for a C10 sub-section; (**a**) as-built map: 0.3 and AI-generated map: 0.7; (**b**) as-built map: 0.85 and AI-generated map: 0.15.

In Figure 7, Area C10 is subdivided into three parts to allow for a better visual interpretation of probability gradient plots. In this example, we consider a refinement to the probability map through the addition of new knowledge. For instance, new knowledge from a recently conducted GPR survey with accurate post-processed data may significantly increase the weight of any source with a utility that agrees with the GPR-detected points. Figure 8 simulates possible outcomes where data are verified first within the AI-generated utility map (left) and second in the as-built map of the utilities (right). In the instance that the GPR data aligned with the AI-generated map, a weight of 0.7 was assigned to the AI-generated map and 0.3 to the as-built map. Alternatively, if the GPR was used to validate the as-built map, weights may be inverted. Here, for example, a weight of 0.15 was assigned to the AI-generated map and 0.85 to the as-built map.

**Figure 9:**
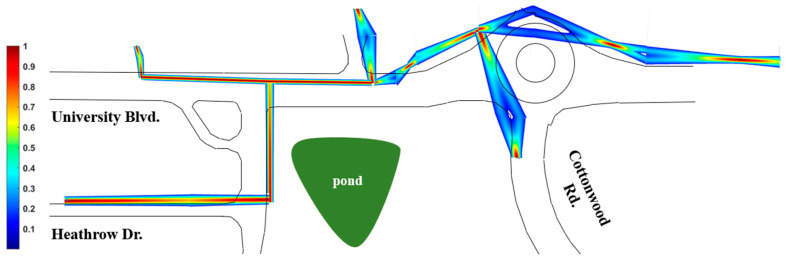
Complete water utility probabilistic map.

Using equal weights of 0.5, Figure 9 shows a full probabilistic color map that considers every set of twin water utility segments in the overlaid map. The color variation helps in the identification of both high and low-confidence areas represented with red and blue colors, respectively. One can observe that regions to the top right of Figure 9 have significantly lower confidence than the region on the left. This information could be useful in guiding further inspections in order to refine the map and eventually prune information that is inaccurate. While initial weights in this example are arbitrary, in the application, weights would be assigned by expert judgment with additional sources likely considered. It may also be eventually possible to automate the weight selection through crowd-sourcing expert judgments across several cases.

It should be noted that our technique maps at the unique class level, and thus does not consider cross-correlations between different utilities. This is attributable to utility codes that typically ensure the physical separation of different utilities. For example, in Iowa (U.S.A.), water and sanitary lines must be separated by 10 ft. Nevertheless, any available information on possible cross-correlations (e.g., two different utilities running in parallel) can be integrated into our method through a knowledge database. This is left to future work.

## 5. Conclusions

The implementation of a probabilistic gradient map for underground utility identification represents a significant advancement in the field of utility mapping and management. Considering the increasing number of accidental utility strikes, the increasing installation of underground utilities, and the resource demands of existing utility investigation methods, the use of a non-invasive and practical methodology can enhance the efficiency, accuracy, and reliability of underground utility detection. This, in turn, reduces risks and costs, minimizes project delays, and ensures the safety of workers and the general public. It also facilitates decision-making during excavation activities.

The use of AI-generated maps has the important potential to quickly map underground utilities by generating asset connections from visual cues and other observed traces of utility construction or past investigations, thereby enhancing the data accuracy and completeness to prevent future accidents. The proposed data fusion processes between the AI-generated utility map and the as-built utility map further enhance the reliability of the information, offering a more comprehensive and precise understanding of the underground utility infrastructure’s underlying uncertainties as shown as a final probabilities map. As a result, decision-makers and utility operators can make more informed choices and minimize potential risks associated with inaccuracies in the data, ultimately leading to safer and more efficient utility management. This includes decisions to conduct additional inspections in low-confidence zones. This method can be particularly convenient for large working areas for which important cost savings could arise by focusing inspection labor, for instance, GPR-based inspections, over particular areas.

While this framework is not designed to replace utility investigation, it offers an opportunity to build increased efficiency into these processes. It can also quickly derive a utility map of any given area with detailed information on uncertainty. This model is useful in preliminary engineering decision-making in construction projects that require an assessment of utility impact density. This approach offers multiple opportunities for savings of time and money, whether in utility investigation operations or associated project development.

## Figures and Tables

**Table 1:** Aboveground features by utility classes (adapted from [12,20]).

Utility	Appurtenances	Locations
Electricity	1, 2, 3, 4, 5, 6, 15, 17	sidewalk, property line, and toward buildings
Sewerage	3, 7, 9, 17	sidewalk, property line, and toward buildings
Stormwater	3, 6, 8, 17	along the road/right of way
Telecom	1, 2, 3, 5, 6, 10, 11, 17	sidewalk, property line, along the road/right of way
Water	3, 12, 13, 16, 17	sidewalk and toward buildings
Natural gas	6, 13, 14, 17	sidewalk and toward buildings

1: pedestal, 2: control box, 3: manhole, 4: transformer, 5: electric poles, 6: trench trail, 7: cleanouts, 8: storm traps, 9: culvert/gate, 10: antenna, 11: closures, 12: fire hydrant, 13: valve cover, 14: gas meter, 15: electric meter, 16: water meter, 17, utility flags.

**Table 2:** Utility hierarchical rules.

Rule Group	Ranking	Rules
		Class: Water Utility
Proximity	1	Connect valve covers in close proximity.
	2	Connect water hydrants to valve covers.
	3	Connect water hydrants along the same lane or on the opposite side of the road in the absence of valve covers.
	4	Remove redundant utility connection in the event of possible closed-circuit connections.
Roadway	5	Connect main utility longitudinally along the roadway.
Downstream feed	6	Connect chillers directly to neighboring buildings.
	7	Connect nearby water-meter manhole and/or shut-off valve directly to the main utility line for utility service connections to buildings.
Class: Electricity utility		
Proximity	1	Connect transformer to nearby electric meters, pedestals, and control boxes.
	2	Connect two transformers in close proximity.
	3	Connect transformer to any outdoor-laid electrical appliances such as a cooling fan.
	4	Connect light poles in close proximity.
Class: Stormwater utility		
Proximity	1	Connect two manholes in close proximity found on the opposite or the same side of the roadway.
	2	Connect only two manholes with the shortest distance and an agreeing slope in the presence of multiple manholes.
	3	Remove redundant utility connection in the event of possible closed-circuit connections.
Roadway	4	Connect utility in the direction of roadway.
Downstream feed	5	Connect a manhole directly to the nearest outfall.
	6	Connect manholes directly to nearby lake, river or ponds.
Class: Sanitary utility		
Proximity	1	Connect two manholes in close proximity found on the opposite or the same side of the roadway.
	2	Connect only two manholes with the shortest distance and an agreeing slope in the presence of multiple manholes.
	3	Remove redundant utility connection in the event of possible closed-circuit connections.
Roadway	4	Connect main utility longitudinally along the roadway.
Downstream feed	5	Connect manholes to neighboring buildings.
Class: Telecom utility		
Proximity	1	Connect two manholes in close proximity found on the opposite or the same side of the roadway.
	2	Connect two pedestals (cabinets) in close proximity found on the opposite or the same side of the roadway.
Class: Gas utility		
Proximity	1	Connect two valve covers in close proximity found on the opposite or the same side of the roadway.
	2	Connect valve covers to nearby gas meter.
	3	Connect a reducing station to any nearby gas meter.
	4	Connect meters with the shortest distance in the presence of multiple gas meters.

**Table 3:** Utility connection mass value determining factors.

Knowledge Source	Factor
As-built map	Map age Construction activities Consistency with observed aboveground asset Validation recency
AI-generated utility map	Asset coordinates precision Assets accessibility Linearity assumption obstacles Utility rule compliance
Extended survey	Recency Mode limitations: (GPR, manhole search, other sensors) Flags and colored markings accuracy

## Data Availability

The raw data supporting the conclusions of this article will be made available by the authors on request.
